# An Acute Reduction in Habitual Protein Intake Attenuates Post Exercise Anabolism and May Bias Oxidation-Derived Protein Requirements in Resistance Trained Men

**DOI:** 10.3389/fnut.2020.00055

**Published:** 2020-04-22

**Authors:** Cassidy T. Tinline-Goodfellow, Daniel W. D. West, Julia M. Malowany, Jenna B. Gillen, Daniel R. Moore

**Affiliations:** Faculty of Kinesiology and Physical Education, University of Toronto, Toronto, ON, Canada

**Keywords:** resistance training, hypertrophy, protein requirements, muscle growth, protein synthesis, stable isotopes, protein oxidation, indicator amino acid oxidation

## Abstract

Protein recommendations for resistance-trained athletes are generally lower than their habitual intakes. Excess protein consumption increases the capacity to oxidize amino acids, which can attenuate post-exercise anabolism and may impact protein requirements determined by stable isotope techniques predicated on amino acid tracer oxidation. We aimed to determine the impact of an acute (5d) reduction in dietary protein intake on post-exercise anabolism in high habitual consumers using the indicator amino acid oxidation (IAAO) technique. Resistance trained men [*n* = 5; 25 ± 7 y; 73.0 ± 5.7 kg; 9.9 ± 2.9% body fat; 2.69 ± 0.38 g·kg^−1^·d^−1^ habitual protein intake) consumed a high (H; 2.2 g·kg^−1^·d^−1^) and moderate (M; 1.2 g·kg^−1^·d^−1^) protein diet while training every other day. During the High protein phase, participants consumed a 2d controlled diet prior to determining whole body phenylalanine turnover, net balance (NB), and ^13^CO_2_ excretion (F^13^CO_2_) after exercise via oral [^13^C]phenylalanine. During the Moderate phase, participants consumed 2.2 g protein·kg^−1^·d^−1^ for 2d prior to consuming 1.2 g protein·kg^−1^·d^−1^ for 5d. Phenylalanine metabolism was measured on days 1, 3, and 5 (M1, M3, and M5, respectively) of the moderate intake. F^13^CO_2_, the primary outcome for IAAO, was ~72 and ~55% greater on the 1st day (M1, *P* < 0.05) and the third day of the moderate protein diet (M3, *P* = 0.07), respectively, compared to the High protein trial. Compared to the High protein trial, NB was ~25% lower on the 1st day (M1, *P* < 0.01) and 15% lower on the third day of the moderate protein diet (M3, *P* = 0.09). High habitual protein consumption may bias protein requirements determined by traditional IAAO methods that use only a 2d pre-trial controlled diet. Post-exercise whole body anabolism is attenuated following a reduction in protein intake in resistance trained men and may require ~3–5d to adapt. This trial is registered at clinicaltrials.gov as NCT03845569.

## Introduction

Dietary protein ingestion acts synergistically with resistance exercise to elevate rates of muscle protein synthesis (MPS) (1, 2) and enhance whole-body net protein balance (3), which over time supports training adaptations such as muscle hypertrophy. The importance of dietary protein for the recovery from and adaptation to resistance exercise is reflected in the generally accepted greater daily recommendations in athletic populations relative to their weight-stable, non-training counterparts (4). For example, traditional nitrogen balance methodology has estimated protein requirements to be ~1.33 g·kg^−1^·d^−1^ (5), which is greater than the current recommended daily allowance of 0.8 g·kg^−1^·d^−1^ (6). However, the safe protein intake (i.e., +2 SD of the estimated average requirement; EAR) by nitrogen balance has also been suggested to be greater in novice weight lifters as compared to trained body builders (i.e., ~1.7 vs. 1.2 g·kg^−1^·d^−1^) (7, 8). This may be related in part to an increased requirement to support muscle damage repair and growth that is typically greatest during the initiation of a resistance training program (9, 10). Viewed through this lens, recent estimates of the protein requirements to maximize whole body protein synthesis on a non-training day by the indicator amino acid oxidation technique (IAAO) in experienced body builders, who would likely have limited hypertrophic potential, of 1.7 g·kg^−1^·d^−1^ (safe intake at the upper 95% confidence interval of 2.2 g·kg^−1^·d^−1^) could be interpreted as curiously high (11). Nevertheless, this EAR is similar to our recent estimates using the IAAO in females (i.e., ~1.5 g·kg^−1^·d^−1^) (12) and men [~1.9 g·kg^−1^·d^−1^; (13)] on a training day. However, these estimated requirements may be irrelevant considering strength athletes habitually consume well in excess of these recommended intakes (i.e., >2 g·kg^−1^·d^−1^) (5), which was indeed the case with the aforementioned IAAO studies that reported mean habitual intakes of ~1.9–2.4 g·kg^−1^·d^−1^ (11–13).

The ability to incorporate dietary amino acids into muscle and body proteins is saturable, with meal protein intakes in excess of ~0.3 g·${\text{kg}}_{\text{BW}}^{-1}$ leading to increased amino acid oxidation (14–16). Overtime, the consumption of protein above the maximal anabolic threshold results in an upregulation of amino acid oxidative capacity as an essential component of the adaptive metabolic demand for protein (17, 18). Studies estimating protein requirements by nitrogen balance typically provide a controlled test diet intake of 5–11d to allow for adaptation to a new intake in light of this adaptive upregulation in amino acid oxidative capacity (19). In contrast, IAAO has been suggested to be valid with only a 2-d pre-trial controlled diet (20), which is a purported strength of the technique permitting a greater number and range of protein intakes to be tested (21). However, the IAAO method relies on the oxidation of a tracer amino acid (the reciprocal of whole-body protein synthesis) (22) and therefore any metabolic adaptations (e.g., upregulation of oxidative enzymes) associated with a high habitual protein intake may impact the oxidation of the indicator or other amino acids, which would translate into an overestimation of protein requirements. This could explain, in part, the relatively high protein recommendations recently reported in resistance trained populations with high habitual protein intakes utilizing IAAO methodology (11–13). Therefore, the purpose of this study was to determine what impact an acute reduction in protein intake has in high habitual protein consumers on ^13^CO_2_ excretion (F^13^CO_2_, the primary outcome of IAAO trials) and the time course for any subsequent metabolic adaptations. In addition, lean mass growth with training has been reported to be supported by a range of protein intakes (i.e., ~1.6 ± 0.4 g·kg^−1^·d^−1^) (23) with moderate levels (i.e., 1.2–1.4 g·kg^−1^·d^−1^) sufficient to support anabolism in novice weightlifters at the onset of training (i.e., ~12wk) (24, 25). Thus, a secondary outcome was to determine how reducing dietary protein intake to a moderate 1.2 g·kg^−1^·d^−1^ from a high intake of 2.2 g·kg^−1^·d^−1^ could support post-exercise anabolism over a 5-d adaptation period. This moderate intake was selected as it represents the IAAO-derived safe intake for non-exercising adults (26), the lower 95% confidence interval from (11) in bodybuilders on a non-training day, is the intake often used for a 2 days adaptation period (12, 27, 28), and is within the range of intakes that have been reported to support lean mass growth with training (23). We hypothesized that, compared to a high protein intake, F^13^CO_3_ would be elevated and whole-body net protein balance would be reduced during post-exercise recovery for up to 5 days after reducing protein intake to a moderate intake in resistance trained athletes accustomed to a high habitual protein diet.

## Methods

### Ethics Statement

All participants were informed of the study purpose, experimental procedures, and potential risks and gave written informed consent. The studies were performed in accordance with the Declaration of Helsinki and the study protocols were approved by the University of Toronto Delegated Research Ethics Board. The study is registered at clinicaltrials.gov (NCT03845569).

### Participants

Potential participants were given a detailed description of the study and potential risks, prior to providing written informed consent. Participants were required to complete the *Physical Activity Readiness Questionnaire* (PARQ+) and provide a complete resistance training (RT) history. Participants completed a self-reported, portion estimated 3-days diet log, which was analyzed (*The Food Processor*, ESHA, Oregon, USA) for determination of habitual dietary intake patterns. To meet out inclusion criteria, eligible participants were required to be a male between 18 and 35 years who had been performing whole-body weight training consistently (≥ 2 training sessions/week) for >1 year, with a habitual protein consumption of 1.9–3.0 g·kg^−1^·d^−1^ (5, 11), and weight-stable over the past month. To ensure an adequate training status, participants were required to meet relative strength-to-weight requirements for bench press (1.0 × bodyweight) and leg press (4.0 × bodyweight) [adapted from Morton et al. (29)]. Exclusion criteria included anabolic drug use, medications known to influence metabolism, or use of creatine or beta-alanine in the past 30 days.

### Experimental Design

Following an overnight fast, participants reported to the Goldring Centre for High Performance Sport at the University of Toronto to have their fat-free mass (FFM), fat mass (FM), and resting metabolic rate (RMR) estimated via air displacement plethysmography (*BodPod*, Cosmed USA Inc., Chicago, IL). Participants then received 75 g of carbohydrate provided as a 1:1 ratio of maltodextrin (*Polycal*; Nutricia, Amsterdam, Netherlands) and sports drink powder (*Gatorade*; PepsiCo), rested for 30 min, and then performed full-body strength testing for determination of one-repetition max (1RM) for the following exercises: bench press, latissimus pulldown; barbell overhead press, seated cable row, leg press, and knee extension.

**Figure 1:**
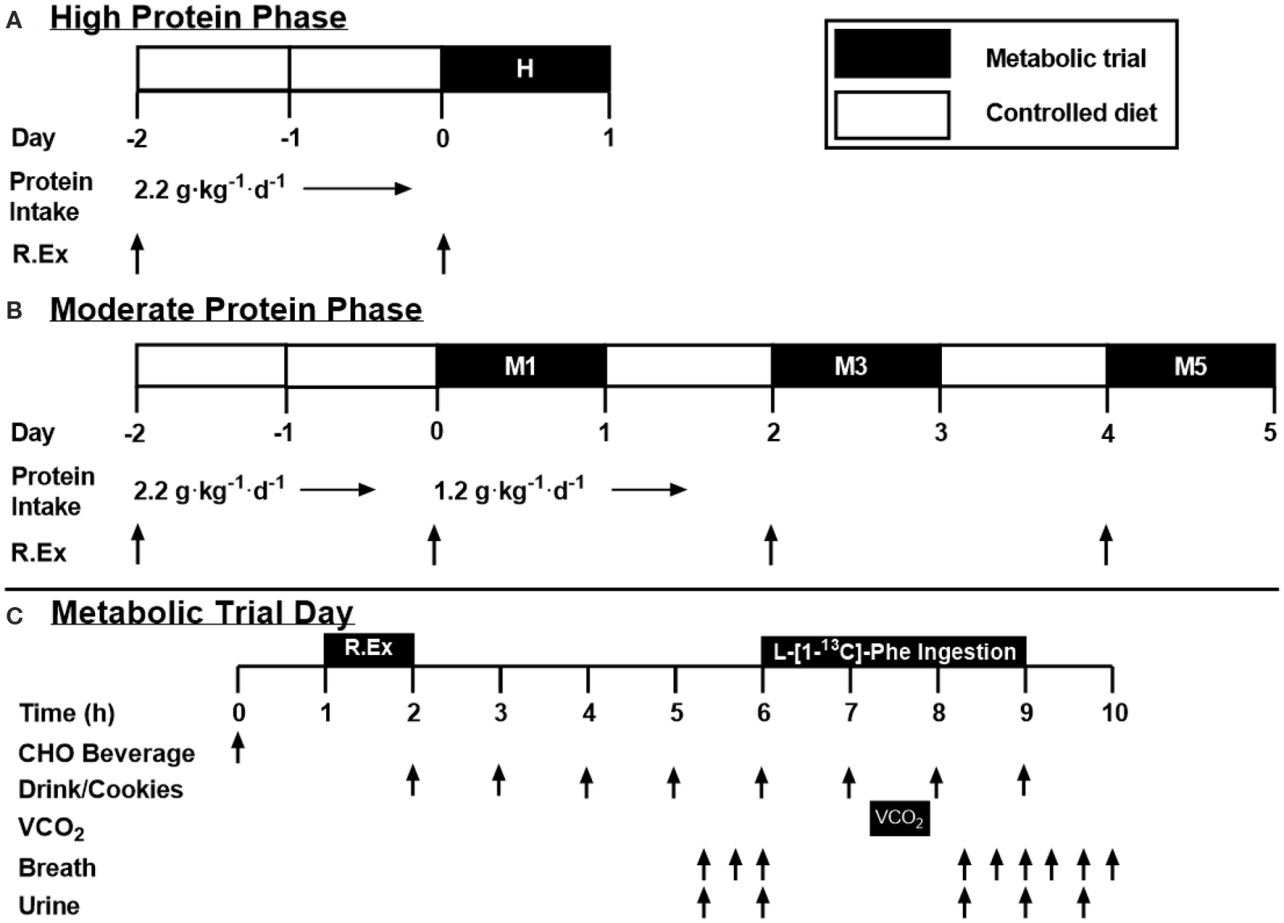
Study design. **(A,B)** High and moderate protein intake phase overview. White bars represent non-trial days, with a controlled diet. Black bars represent a metabolic trial day. **(C)** Metabolic trial day overview. Test drinks provided 1/12th of dietary requirement each, with ingestion of the tracer (L-[1-^13^C] phenylalanine) beginning with drink five. R.Ex, resistance exercise; VCO_2_, volume of expired CO_2_ collected via indirect calorimetry; CHO, Carbohydrate.

In a cross-over design, participants completed high (H; 2.2 g protein·kg^−1^·d^−1^) and moderate (M; 1.2 g protein·kg^−1^·d^−1^) protein intake phases separated by at least 1 week (summarized in Figures 1A,B, respectively). The protein level for M was selected to reflect the lower boundary protein recommendations by consensus statements (4) and the lower 95%CI by IAAO (11) whereas H represented the upper 95%CI of recently determined recommendations by IAAO (11) and approximated the average reported protein intake of resistance-trained athletes (i.e., ~2.1 g·kg^−1^·d^−1^) (5). These protein levels would also span recent estimates of the estimated average requirement (EAR) that has been suggested to maximize lean mass growth with training (23) and, in our hands, post-exercise whole body anabolism (12, 13). The H phase consisted of a 2 days commercially available prepackaged food diet providing total energy that was equivalent to 1.5 × RMR, 2.2 g protein·kg^−1^·d^−1^, 3–5 g carbohydrate·kg^−1^·d^−1^, and the remaining calories in the form of dietary fat followed by a metabolic trial on Day 3 (see below for details). Protein in these pre-packaged diets was a mixture of animal and plant-based protein sources. The High phase was 2 days in length as it was meant to approximate the population's habitual dietary protein intake and therefore would not require a substantial (if any) adaptation period. On Day 1, participants performed whole-body resistance exercise consisting of four sets of 10 repetitions at 75% of their 1RM with 2 min rest between sets. Push and pull supersets were performed for upper body exercise with bench press paired with latissimus pulldown, and overhead press paired with seated cable row, whereas leg press and leg extensions were performed in isolation. On Day 3, participants performed a metabolic trial (described below). The M phase lasted 7 days, with a metabolic trial on Days 1, 3, and 5 following the reduction of dietary protein intake (M1, M3, and M5). Dietary intake was controlled throughout the whole phase, providing either 2.2 g·kg^−1^·d^−1^ of protein (Days−2 and−1), or 1.2 g·kg^−1^·d^−1^ (Days 1 through 5; Figure 1), with the same amount of energy, carbohydrate and fat as H. Whole-body resistance exercise was performed on Days−2, 1, 3, and 5. With this design, we were able to determine if high habitual protein intake influences protein oxidation upon a decrease in protein intake (H1 vs. M1), as well as determine the number of days required for adaption to a lower protein intake (M1 vs. M3 vs. M5). Further, this design informed us as to whether the standard IAAO method protocols (11, 12, 22) may reflect–as a result of a short, 2 days control period prior to metabolic trials–the metabolic state of individuals habitually consuming a high protein diet.

### Metabolic Trials

The metabolic trial is depicted in Figure 1C and is consistent with previous work from our laboratory (12, 30). Briefly, on metabolic trial days, 1 h prior to engaging in RT, participants consumed a beverage containing 75 g of carbohydrate. Immediately following the whole-body resistance exercise, participants consumed the first of eight isocaloric and isonitrogenous hourly meals in the form of test drinks and protein-free cookies (31). The test drinks provided a total of two thirds of the participants' daily requirement at 1.2 or 2.2 g·kg^−1^·d^−1^ of protein, 4 g·kg^−1^·d^−1^ of carbohydrate, and the remainder of calories from dietary fat, for a total of 1.5 × RMR. The test drinks provided dietary amino acids in form of crystalline amino acids (Ajinomoto North America, Inc., Raleigh, NC) modeled on egg protein with the exception of phenylalanine (the indicator amino acid) and tyrosine, which were provided at 30.5 and 40 mg·kg^−1^·d^−1^, respectively (11). Tyrosine was provided in excess to ensure the labeled carboxyl group of dietary phenylalanine was partitioned to either protein synthesis or oxidation, and not retained in the body tyrosine pool (32, 33). As well as the crystalline amino acids, the test drinks contained protein-free powder (*PFD*; Mead Johnson, Evansville, IN), flavoring crystals (*Tang*; Kraft, Don Mills, Canada), maltodextrin (*Polycal*; Nutricia, Amsterdam, Netherlands), and grape seed oil (President's Choice; Loblaw Companies, Brampton, Canada). Additionally, the fifth meal contained a priming dose of NaH^13^CO_3_ (0.176 mg·kg^−1^) and additional L-[1-^13^C] phenylalanine (1.80 mg·kg^−1^ total; 1.22 mg·kg^−1^ plus an additional 0.66 mg·kg^−1^). Subsequently, 1.22 mg·kg^−1^ of L-[1-^13^C] phenylalanine was ingested in each hourly meal to maintain isotopic steady state until the end of the metabolic trial. Following the metabolic trial, participants were sent home with food to fulfill the remaining one third of their daily dietary requirements.

### Breath and Urine Sample Collection and Analysis

Prior to the initiation of the oral tracer ingestion (hourly meal five), three breath samples were taken at 15-min intervals, and two urine samples were collected at 30-min intervals to determine baseline (i.e., background) ^13^CO_2_ and L-[1-^13^C]phenylalanine enrichments, respectively. The steady state production of CO_2_ was measured over a 20-min period via indirect calorimetry (iWorx GA-300, Dover, NH), ~30 min after the fifth drink. Breath and urine samples were collected isotopic and metabolic steady-state at 30-min intervals beginning 2.5 h after the onset of tracer ingestion (i.e., 2.5 h following test drink five). Breath ^13^CO_2_ enrichment was measured by continuous-flow isotope ratio mass spectrometry and urinary L-[1-^13^C]phenylalanine was measured by liquid chromatography tandem mass spectrometry, as described previously (12).

### Tracer Kinetics

Tracer kinetics were determined according to previous studies (30, 34), Briefly, phenylalanine flux (PheRa; μmol·kg^−1^·h^−1^) was calculated using the following equation:

$$\begin{array}{l} \text{PheRa}=i\;•\left(\frac{{\text{E}}_{\text{i}}}{{\text{E}}_{\text{u}}}\right)-I \end{array}$$

Where *i* represents the rate of L-[1-^13^C] phenylalanine ingestion (μmol·kg^−1^·h^−1^). E_i_ and E_u_ are the isotopic enrichments as a mole fraction in atom percent excess (APE) of the test drink and urinary phenylalanine, respectively, at isotopic steady-state. *I* is the rate of L-phenylalanine ingestion (μmol·kg^−1^·h^−1^).

The rate of ^13^CO_2_ appearance in the breath (fraction of expired ^13^CO_2_, F^13^CO_2_; μmol·kg^−1^·h^−1^) was calculated using the following equation:

$$\begin{array}{l} F^{13}CO_{2}=\;\left(V_{CO2}\right)\;.\left(E_{CO2}\right).\left(44.6\right)\;.\left(60\right)\;.BW^{-1}\;.\;\left(0.82\right)\;.\left(100\right) \end{array}$$

Where V_CO2_ is equal to the volume of CO_2_ produced (mL·min^−1^); E_CO2_ is the breath ^13^CO_2_ enrichment at isotopic steady state (APE); BW is bodyweight in kilograms. 44.6 μmol·kg^−1^·h^−1^ and 60 min·h^−1^ were constants used to convert F^13^CO_2_ to μmol·h^−1^. A factor of 0.82 was used to correct for CO_2_ retained in the bicarbonate pool in the fed state (35).

Phenylalanine oxidation (PheOx; μmol·kg^−1^·h^−1^) was calculated using E_u_ as an estimate of intracellular enrichment (36) using the equation:

$$\begin{array}{l} \text{PheOx}={\text{F}}^{13}CO_{2\;}•\left(\frac{1}{E_{u}}-\frac{1}{E_{i}}\right)\times 100 \end{array}$$

Using standard steady state equations (34), whole-body protein breakdown was assumed to reflect PheRa whereas non-oxidative phenylalanine disposal (NOPD; estimate of protein synthesis) was calculated as the difference between PheRa and PheOx. Whole-body phenylalanine net balance (NB) was calculated as the difference between NOPD and Ra.

### Statistical Analysis

Statistical analyses were completed using GraphPad Prism (version 8.00, GraphPad Software, San Diego, CA) with significance set at *P* < 0.05. A one-way repeated measures ANOVA was performed on data from F^13^CO_2_, phenylalanine rate of appearance (i.e., flux; PheRa), phenylalanine oxidation (PheOx), and phenylalanine net balance (NB). *Post-hoc* pairwise analysis was performed using a Holm-Sidak correction. Cohen's *d* was calculated using an equation for paired samples (37, 38):

$$\begin{array}{l} d=\frac{\left(M_{1}-M_{2}\right)}{SD_{pooled}} \end{array}$$

Where *M*_1_ and *M*_2_ are the means being compared, and *SD*_pooled_ is the pooled standard deviation for the ANOVA, calculated by taking the square root of the residual mean square (MS_residual_). Standardized difference thresholds were defined as very small < 0.2, small < 0.5, moderate < 0.8, and large ≥ 0.8. Data are expressed as mean ± standard error (SE).

## Results

### Participant Characteristics

Eight participants were screened for eligibility and five completed all trials and were included in data analysis. Three participants did not complete any metabolic trials due to dropout due to time commitments (*n* = 2) or inability to meet strength-to-bodyweight requirements (*n* = 1). Three participants were randomized to complete the high protein phase first and two were randomized to complete the moderate protein phase first. Participant characteristics are shown in Table 1.

**Table 1:** Physical characteristics and habitual dietary intake of participants^a^.

**Characteristics**	**Values**
Age, y	25 ± 7
Body weight, kg	73.1 ± 5.7
Height, cm	180 ± 5
Body Fat, %	10.0 ± 2.9
FFM, kg	65.8 ± 5.8
Bench Press 1RM, kg	98.0 ± 11.0
Bench Press 1RM, kg/BW	1.34 ± 0.08
Leg Press 1RM, kg	315 ± 38.2
Leg Press 1RM, kg/BW	4.31 ± 0.25
**Habitual protein intake**	
g·kg^−1^·d^−1^	2.7 ± 0.4
% of daily kcal	28.4 ± 5.3

a *All values are means ± SD*.

*FFM, fat free mass; 1RM, 1 repetition maximum; BW, body weight*.

### Phenylalanine Flux

**Figure 2:**
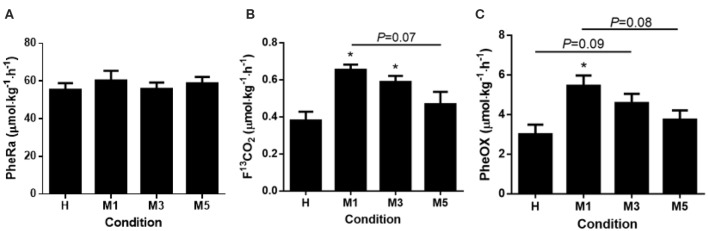
Phenylalanine metrics. **(A)** Phenylalanine rate of appearance (PheRa, Flux) had no effect for time. **(B)** There was a main effect of condition for fraction of expired ^13^CO_2_ (F^13^CO_2_). F^13^CO_2_ was significantly elevated from H to M1 (*d* = 2.58), and from H to M3 (*d* = 1.96). There was a trend for M1 to be elevated compared to M5 (*d* = 1.75, *P* = 0.07). **(C)** Phenylalanine oxidation (PheOx) displayed a main effect of condition and was significantly elevated at M1 compared to H (*d* = 2.56). There was a trend for M3 to be significantly higher than H (*d* = 1.66, *P* = 0.09). There was a trend for M5 to be lower than M1 (*d* = 1.78, *P* = 0.08). The effect of condition was determined using a repeated measures one-way ANOVA, with a Holm-Sidak *post-hoc* for pairwise comparisons. ^*^Significantly different from H (*P* < 0.05). Data are means ± SE.

There was no change in phenylalanine flux (PheRa) across the metabolic trials (57.6 ± 3.7 μmol·kg^−1^·h^−1^; *P* = 0.23, Figure 2A).

### F^13^CO_2_ Excretion

There was a main effect of condition for F^13^CO_2_ (*P* < 0.01; Figure 2A). M1 (0.65 ± 0.03 μmol·kg^−1^·h^−1^, *d* = 2.58) and M3 (0.59 ± 0.03, *d* = 1.96) were >H (0.38 ± 0.05, *P* < 0.05) respectively, whereas M5 (0.47 ± 0.07) was not different from H (*d* = 0.83; *P* = 0.38; Figure 2B). There was a trend (*P* = 0.07) toward significance between M1 and M5 (*d* = 1.75). Despite a large effect (*d* = 1.13), there was no statistical difference between M3 and M5 (*P* = 0.27).

### Phenylalanine Oxidation

There was a main effect of condition for PheOx (*P* < 0.01; Figure 2C). M1 (5.5 ± 0.5 μmol·kg^−1^·h^−1^, *d* = 2.56) was >H (3.0 ± 0.5, *P* < 0.01). There was a trend for M3 (4.6 ± 0.5, *d* = 1.66, *P* = 0.09) being >H. Despite a moderate effect, was no statistical difference between M5 (3.7 ± 0.5, *d* = 0.79) and H (*P* = 0.44). Three was a trend (*P* = 0.08) for M1 being >M5 (*d* = 1.78). Despite large effects, there no statistical differences between M1 and M3 (*d* = 0.91, *P* = 0.44) and M3 and M5 (*d* = 0.87, *P* = 0.44).

### Phenylalanine Net Balance

**Figure 3:**
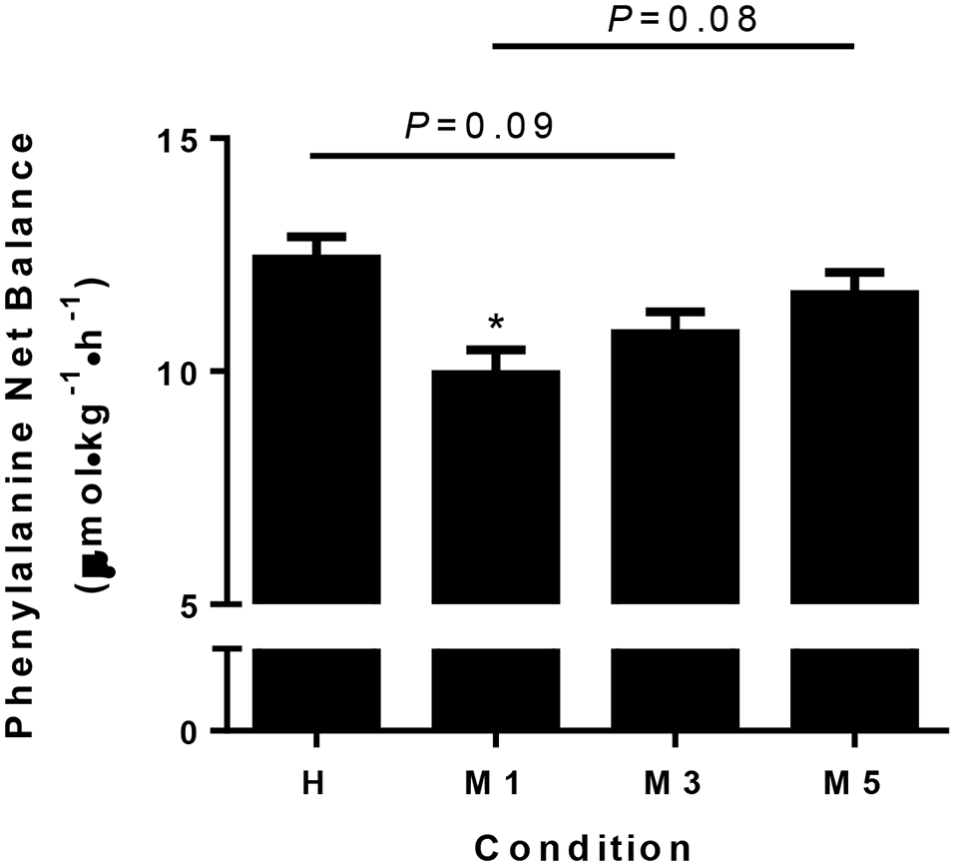
Phenylalanine net balance. Whole body phenylalanine net balance is significantly decreased at M1 compared to H (*d* = −2.57), with a trend for M3 to be less than H (*d* = −1.68, *P* = 0.09). There was a trend for M5 to be greater than M1 with a large effect size (*d* = 1.78, *P* = 0.08), suggesting adaptation to the moderate protein intake may take 5d. The effect of condition was determined using a repeated measures one-way ANOVA, with a Holm-Sidak *post-hoc* for pairwise comparisons. ^*^Significantly different from H (*P* < 0.05). Data are means ± SE.

There was a main effect of condition for NB (*P* < 0.01; Figure 3) as it was lower in M1 (9.9 ± 0.5 μmol·kg^−1^·h^−1^) compared to H (12.4 ± 0.5, *d* = −2.57, *P* < 0.01,). There was a trend (*P* = 0.09) toward significance for M3 (10.8 ± 0.5, *d* = −1.66) being lower than H. There was a moderate effect (*d* = –0.79) that was not statistically significant (*P* = 0.44) for M5 (11.7 ± 0.5) to be lower than H. There was a trend (*d* = –1.78, *P* = 0.08) for M1 to be lower than M5, but there were no statistical differences between M1 and M3 (*d* = –0.91, *P* = 0.44) or M3 and M5 (*d* = –0.87, *P* = 0.44).

## Discussion

Dietary amino acids consumed in excess of their ability to be incorporated into *de novo* muscle and body protein synthesis are irreversibly oxidized (22), representing the primary contributor to the adaptive metabolic demand for amino acids (39). We demonstrate that reducing dietary protein intake in habitually high protein consumers from a standardized 2.2 g·kg^−1^·d^−1^ to a moderate 1.2 g·kg^−1^·d^−1^ resulted in a reduction in whole-body net protein balance during recovery from resistance exercise for up to 3 days, although greater durations (i.e., ≥5 days) may be needed for full adaptation. Importantly, this reduced net balance was not related to any accommodation in whole body protein breakdown (i.e., PheRa) but rather was the result of an elevated excretion of our indicator amino acid (i.e., F^13^CO_2_), suggesting that metabolic adaptation in high habitual protein consumers requires greater durations than the traditional 2 days adaptation period employed in IAAO studies (11, 12, 20, 30).

The principle of the IAAO is the indicator amino acid (phenylalanine) is partitioned toward oxidation when there is a limitation in a single or multiple amino acids (with the exception of tyrosine) to support its use for protein synthesis. Seminal work in the development of the IAAO method demonstrated that 8 h to 9 days of adaptation to various intakes of single amino acids (i.e., lysine and phenylalanine) had no influence on L[1-^13^C]phenylalanine oxidation when consuming the remaining amino acids at a constant protein intake equivalent to 1.0 g·kg^−1^·d^−1^ (33, 40). This was suggested to represent a strength of IAAO methodology to determine amino acid and/or protein requirements given the apparently short adaptation time required relative to the traditional nitrogen balance technique (i.e., ≤ 8 h vs. ≥5 days) (21). However, it was subsequently demonstrated that although the amount of protein (0.8, 1.4, or 2.0 g·kg^−1^·d^−1^) consumed in the 2 days prior to an IAAO metabolic trial (providing 1.0 g·kg^−1^·d^−1^) had no discernable pattern of influence on F^13^CO_2_ there was significant variability in phenylalanine flux and oxidation in a small subset (*n* = 5) of healthy adults consuming a habitual intake of ~1.0–1.5 g protein·kg^−1^·d^−1^ (20). As a result, a 2-days adaptation period of controlled protein intake has been assumed to be sufficient prior to metabolic trials using IAAO methodology and have therefore been employed in studies estimating protein requirements in active populations who traditionally consume protein at intakes several fold that of the current RDA (5, 11, 41). In contrast to previous research, our F^13^CO_2_ results suggest that high habitual protein consumers require at least 3 days and perhaps up to 5 days to adapt to a lower controlled dietary protein intake. These data would be consistent with an increased amino acid oxidative capacity with excess protein consumption (17, 18, 42) that in the case of the branched chain amino acids may be mediated by increased expression and/or activity of the rate controlling enzyme branched chain α-ketoacid dehydrogenase complex (43, 44). Therefore, our results demonstrate the traditional 2-days pre-trial controlled diet that is typical of most IAAO studies would be insufficient to ensure proper metabolic adaptation in populations who habitually consume high, and arguably excessive, protein intakes, which subsequently could result in a biased overestimation of true protein requirements.

The indicator amino acid is provided in excess of its metabolic requirement to ensure it is always partitioned toward oxidation, which would be lowest at a sufficient protein/amino acid intake. It is notable therefore that F^13^CO_2_ (the most direct estimate of oxidation of the indicator amino acid) (21) at a surfeit protein intake (i.e., ≥2.2 g·kg^−1^·d^−1^) is similar between the present study and previous studies estimating the protein needs of high habitually consuming resistance-trained athletes (Table 2). The similar F^13^CO_2_ observed in the present study compared to previous work (Table 2) suggests that an equivalent capacity to utilize the indicator amino acid to support protein synthesis (the reciprocal of F^13^CO_2_) was obtained across the present and previous studies at these surfeit protein intakes. We also observed similar F^13^CO_2_ on M3 (i.e., after a traditional 2-d dietary adaptation period) in the present study as the estimated F^13^CO_2_ at 1.2 g·kg^−1^·d^−1^protein consumption in previous studies (11, 12). The similarity in the ratio of F^13^CO_2_ at 1.2 and 2.2 g·kg^−1^·d^−1^ protein intake across studies (i.e., ~1.6–1.9) suggests the habitually high intakes of these populations had a similar impact on the ability of lower protein intakes to support protein synthesis. Although F^13^CO_2_ was not statistically different after 5 days of adaptation to a moderate (1.2 g·kg^−1^·d^−1^) as compared to high (2.2.g·kg^−1^·d^−1^) protein intake it remained elevated by ~23%, which would be generally consistent with a requirement of up to 7 days to down-regulated leucine oxidation by ~35% after a ~1 g·kg^−1^·d^−1^ reduction in dietary protein (45). Taken together, the attenuation in F^13^CO_2_ after initiation of a 2 days moderate 1.2 g·kg^−1^·d^−1^ protein intake could indicate that previous estimates of the protein requirements in strength-trained populations by IAAO may be overestimated by at least ~25% (i.e., M3 vs. M5) (12, 13) and potentially greater with higher [i.e., 1.5–1.6 g·kg^−1^·d^−1^ (11, 46)] adaptation intakes. Therefore, in our hands, an insufficient dietary protein adaptation period in high habitual protein consumers would have little impact on F^13^CO_2_ at surfeit protein intakes but could result in a rightward shift in the bi-phase-determined breakpoint (i.e., EAR) and an overestimation of true protein requirements when using the IAAO.

**Table 2:** Comparison of tracer kinetics in resistance trained men between IAAO-style studies; Data are means ± SD^a^.

	**Test protein intake (g·kg^−1^·d^−1^)**							
	**Present study**				**Mazzulla et al**. **(****13****)**		**Bandegan et al**. **(****11****)**	
	**2.2 (H)**	**1.2 (M1)**	**1.2 (M3)**	**1.2 (M5)**	**2.2**	**1.2**	**2.2**	**1.2**
F^13^CO_2_ (μmol·kg^−1^·h^−1^)^b^	0.38 ± 0.11	0.65 ± 0.06	0.59 ± 0.07	0.47 ± 0.15	0.33	0.52	0.40	0.75
Habitual Protein Intake (g·kg^−1^·d^−1^)	2.7 ± 0.4	2.7 ± 0.4	2.7 ± 0.4	2.7 ± 0.4	2.3 ± 0.6	2.3 ± 0.6	2.4 ± 0.8	2.4 ± 0.8

a *Used reported study average for Mazzulla et al. (13) and Bandegan et al. (11)*.

b *Determined using WebPlot Digitizer online (https://automeris.io/WebPlotDigitizer/)*.

Resistance training-induced increases in FFM can be supported by a range of dietary protein intakes (23). Data from infusion trials suggests post-exercise skeletal muscle anabolism can be maximized with 4–5 meals per day providing ~0.3 g·kg^−1^ per meal or the equivalent (assuming a balanced distribution) of ~1.2–1.5 g·kg^−1^·d^−1^ (14, 16, 47). The present study demonstrates that reducing protein overconsumption in high habitual consumers results in an acute attenuation of whole-body net balance that approaches the anabolic potential of a high (excessive) dietary protein intake by ~5 days (M5). Importantly, phenylalanine flux was not influenced by protein intake suggesting there was no maladaptive down-regulation of whole-body protein metabolism during this acute adaptation phase. Thus, we argue that reductions in whole-body phenylalanine net balance primarily reflect an adaptation to amino acid oxidative capacity toward a more efficient dietary utilization of amino acids (i.e., a larger proportion used for protein synthesis vs. oxidation). Indeed, early adaptations (12 weeks) to resistance training in which muscle growth rate would be greatest (9) are supported by protein intakes of 1.2–1.4 g·kg^−1^·d^−1^ (24, 25), which would be substantially lower than the habitual consumption in this population (5). These findings suggest resistance trained men habitually consuming a high protein diet may be able to maintain whole body anabolism on a moderate protein intake provided adequate time to adapt to this lower intake. Conversely, the high habitual consumption in these populations would ultimately beget a reciprocally high metabolic demand for dietary amino acids to offset the oxidative losses, which may be reflected in the recent and arguably high estimated protein requirements of resistance trained populations by IAAO (11, 13). This increased metabolic demand for dietary amino acids would be consistent with the well-known diurnal cycling of body nitrogen (i.e., fasted state losses countered by fed state gains), which has a greater amplitude at high (i.e., ~2.1 g·kg^−1^·d^−1^) compared to moderate (i.e., ~1.6 g·kg^−1^·d^−1^) intakes (48, 49). In addition, higher protein intakes may also be needed to support the post-prandial utilization of dietary amino acids for muscle protein synthesis given that splanchnic extraction is greater with high protein diets while mixed and myofibrillar protein synthesis is attenuated after consumption of a moderate protein intake (42, 50). Therefore, future research should determine the impact of a reduction in habitual dietary protein intake on post-exercise rates of muscle protein synthesis in the fed state.

The present study utilized a relatively small sample size (*n* = 5) but one that is in line with seminar nitrogen balance research identifying protein requirements in active populations (*n* = 6–7/group) (7, 17, 51, 52) and previous IAAO method validation studies (*n* = 4–6) (20, 53, 54). This apparently small sample size could reduce statistical power and predispose our study to potential type II errors, which could be evident by the statistical trends we observed in some comparisons. To circumvent this, we also reported effect sizes for the comparisons, which revealed moderate to large effects for the statistical trends. Given the similar responses we observed across our population and the physiologically sensible changes in protein metabolism that would be consistent with a successful adaptation to a lower protein intake, we do not believe our conclusions would be altered by a greater sample size but rather statistical trends would merely reach the α = 0.05 threshold to be deemed significant.

In conclusion, our findings suggest that reducing dietary protein in high habitual protein consumers decreases whole body net balance acutely for at least 3 days and perhaps up to 5 days. Our data are consistent with previous data in sedentary individuals (19, 45) showing that, following an acute reduction in dietary protein intake, metabolic adaptations require ≥5 days to adapt to the new intake. Collectively, the 2 days adaptation period prior to metabolic trials that is typical of IAAO protocols may be inadequate when studying populations with high habitual protein intakes and lead to an erroneous overestimation of true protein requirements to support whole body protein synthesis. However, consistent with previous findings in novice weight lifters (24, 25), whole body anabolism may be supported by a moderate (1.2 g·kg^−1^·d^−1^) protein intake in otherwise high habitual protein consuming resistance trained men provided sufficient time is allotted to adapt to this lower intake.

## Data Availability Statement

The datasets generated for this study are available on request to the corresponding author.

## Ethics Statement

The studies involving human participants were reviewed and approved by Health Sciences Research Ethics Board, University of Toronto. The patients/participants provided their written informed consent to participate in this study.

## Author Contributions

CT-G, DW, JG, and DM designed the research. CT-G, DW, and JM conducted the research. CT-G, DW, and JG analyzed the data. CT-G, DW, and DM wrote the paper with input from JM and JG. DM has primary responsibility for final content. All authors have read and approved the final manuscript.

## Conflict of Interest

The authors declare that the research was conducted in the absence of any commercial or financial relationships that could be construed as a potential conflict of interest. The reviewer KT declared a past collaboration with one of the authors DM to the handling editor.
